# Decreased Eccentric Exercise-Induced Macrophage Infiltration in Skeletal Muscle after Supplementation with a Class of Ginseng-Derived Steroids

**DOI:** 10.1371/journal.pone.0114649

**Published:** 2014-12-11

**Authors:** Szu-Hsien Yu, Chih-Yang Huang, Shin-Da Lee, Ming-Fen Hsu, Ray-Yau Wang, Chung-Lan Kao, Chia-Hua Kuo

**Affiliations:** 1 Laboratory of Exercise Biochemistry, University of Taipei, Taipei City, Taiwan, Republic of China; 2 Department of Leisure Industry and Health Promotion, National Ilan University, Yilan County, Taiwan, Republic of China; 3 Institute of Basic Medical Science, China Medical University, Taichung City, Taiwan, Republic of China; 4 Department of Health and Biotechnology, Asia University, Taichung City, Taiwan, Republic of China; 5 Department of Physical Therapy and Assistive Technology, National Yang-Ming University, Taipei City, Taiwan, Republic of China; Chang-Gung University, Taiwan

## Abstract

Dammarane steroids (DS) are a class of chemical compounds present in *Panax ginseng*. Here, we evaluated the effect of 10 weeks of DS supplementation on inflammatory modulation in the soleus muscle following eccentric exercise (EE)-induced muscle damage (downhill running). Eighty rats were randomized into 4 groups of DS supplementation (saline, 20, 60, 120 mg/kg body weight). Inflammatory markers were measured at rest and again 1 h after EE. At rest, NFκB signaling, TNF-alpha and IL-6 mRNAs, 3-nitrotyrosine, glutathione peroxidase, and GCS (glutamylcysteine synthetase) levels were significantly elevated in the skeletal muscle of DS-treated rats in a dose-dependent manner. Additionally, there were no detectable increases in the number of necrotic muscle fibers or CD68^+^ M1 macrophages. However, muscle strength, centronucleation, IL-10 mRNA expression, and the number of CD163^+^ M2 macrophages increased significantly over controls with DS treatment in rat soleus muscle. Under EE-challenged conditions, significant increases in muscle fiber necrosis, CD68^+^ M1 macrophage distribution, and 3-nitrotyrosine were absent in rats that received low and medium doses (20 and 60 mg/kg) of DS treatment, suggesting that DS possess anti-inflammatory action protecting against a muscle-damaging challenge. However, this protective activity was diminished when a high dose of DS (120 mg/kg) was administered, suggesting that DS possess hormetic properties. In conclusion, our study provides new evidence suggesting that DS is an ergogenic component of ginseng that potentiate inflammation at baseline but that produce anti-inflammatory effects on skeletal muscle following muscle-damaging exercise. Furthermore, high doses should be avoided in formulating ginseng-based products.

## Introduction

Inflammation, an innate immune response, plays a key role in eliminating unhealthy cells generated by various adverse conditions [1]; this process is essential for the regeneration of new muscle fibers after damage [2]. At the cellular level, NFκB activation in damaged skeletal muscle facilitates an inflammatory response, which increases interleukin-1beta (IL-1β), tumor necrosis factor alpha (TNF-α), interleukin-6 (IL-6), inducible nitric oxide synthase (iNOS), and cyclooxygenase-2 (COX-2) [3]. This response facilitates further clearance of damaged fibers by communicating with surrounding mononuclear cells. Two phenotypes of mononuclear cells, M1 (CD68^+^ population) and M2 (CD163^+^ population) macrophages, are known to orchestrate this reconstructive program in damaged skeletal muscle. During the early phase, M1 macrophages are recruited to damaged muscle fibers, and free radicals are released to lyse cells in inflamed tissue [4]. This process makes room for tissue repopulation. Changes in redox state after the burst of oxidative free radicals, followed by increased antioxidant production, appears to be essential for the recruitment of stem cells to the damaged site for cell regeneration within a controlled period of time [5]. During the late phase, M1 macrophages are replaced by M2 macrophages to drive a protracted, tissue-specific differentiation. Release of interleukin-10 (IL-10) from M2 macrophages is associated with inhibition of M1 macrophages to end the first destructive phase [4], [6].

Inflammation plays a significant part in the remodeling and adaptation of skeletal muscle in response to physical challenge [7]. In young healthy animals, local anti-inflammatory treatment delays skeletal muscle fiber differentiation after an over-load lesion [8], blocks stem cell proliferation in EE-challenged muscle in humans [9], slows hypertrophy in a rat synergist ablation model [10], [11], and decreases recovery of force development in muscle injury [12]. These previous findings suggest that inflammatory modulation plays a crucial role in muscle adaptation against physical challenges.

Dammarane steroids (DS), a class of steroids present in many ginseng species, have been shown to have an anti-inflammatory activity [13], [14]. However, other studies have reported an increase in cell death after DS treatment in cultured cells [15], [16], suggesting that DS possess pro-inflammatory properties [17]. The underlying mechanisms behind this paradox are not clearly known. Ginseng has been used for thousands of years as a popular supplement to enhance stamina and coping capacity against physical fatigue. However, most studies have not controlled for the steroid profile of ginseng extract; this can be a major confounder and may be responsible for the inconsistent results observed among studies [18], [19]. The steroid profile of ginseng varies with species and cultivating season [20], [21]. In this study, the effects of the ginseng-derived steroids DS on inflammation and strength were examined in rat skeletal muscle after 10 weeks of oral supplementation. Eccentric exercise is known to cause fiber injury and elicit an inflammatory response in contracted skeletal muscle. Inflammation-associated markers were measured in skeletal muscle at rest and 1 h following an acute bout of downhill running, which consists of eccentric muscle contraction, in rats that received various doses of DS supplementation.

## Materials and Methods

### Dammarane steroids (DS)

**Figure 1:**
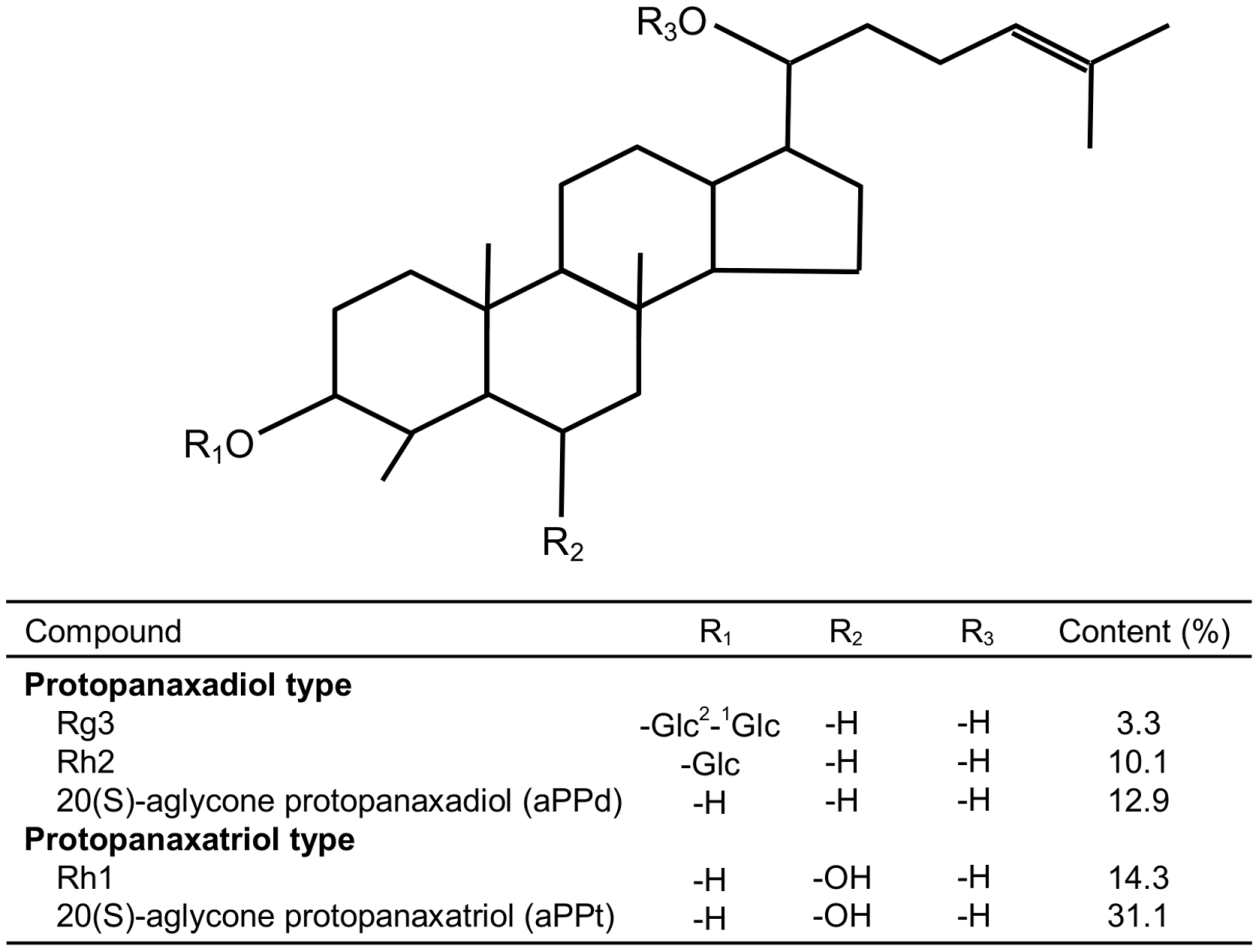
Chemical structure of dammarane steroids from *Panax ginseng*.

The chemical structures and content of DS (ChemSpider ID: 7827637) are shown in Fig. 1, and they were obtained from Pegasus Inc. (Vancouver, Canada). DS (20 mg) was dissolved in 10 ml methanol as test solution and purified by high performance liquid chromatography (HPLC) on a C-18 Silica-based reversed phase HPLC column (Agilent ODS-C18, 5 µm, 4.6×150 mm). The mobile phase was a 2∶4∶2∶1 mixture of chloroform, ethyl acetate, methanol and water, and the separation conditions were as follows: 120 ml mobile phase was injected for each separation, the flow rate was 1 ml/min, and was eluted with a retention time of 120 min. The DS was confirmed by comparison of the physical and spectral data using above process with reference standard solution containing 1.5 mg of Rh1 (C_36_H_64_O_9_), Rg3 (C_30_H_54_O_3_), Rh2 (C_36_H_64_O_8_), 20(S)-aglycone protopanaxadiol (aPPd) (C_30_H_54_O_3_) and 20(S)-aglycone protopanaxatriol (aPPt) (C_30_H_54_O_4_). The content of DS powder contained Rh1 (14.3%), Rg3 (3.3%), Rh2 (10.1%), aPPd (12.9%) and aPPt (31.1%).

### Animals

Eighty male Sprague Dawley (SD) rats (body weight 410±10 g) were obtained from LASCO Corporation (Taipei, Taiwan) at 4 months of age and maintained in the Animal Center of University of Taipei (Taipei, Taiwan). Two animals were housed per cage with standard laboratory chow (PMI Nutrition International, Brentwood, MO, USA) and tap water *ad libitum*. All animals were kept in an animal room with a 12/12 h light/dark cycle, 22±2°C and 50% relative humidity. This study was approved by the Animal Care and Use Committee at University of Taipei (approval number 9807001), and according to the ethical rules in the National Institutes of Health Guide for the Care and Use of Laboratory Animals. Sacrifice was performed under chloral hydrate anesthesia, and efforts were made to prevent or minimize suffering.

### Experimental design

A 2×2 factorial design (supplemental dosage and exercise challenge) was used in this study. Following a 7-day acclimatization to the housing environment at University of Taipei, the 80 rats were randomized by weight into 4 groups. Each group was assigned one of 4 daily DS doses: saline (Control, N = 20) and 20 (DS20, N = 20), 60 (DS60, N = 20), or 120 mg/kg BW (DS120, N = 20). DS were dissolved in saline by vortexing immediately before oral intubation. Equal volumes of DS and saline were delivered daily by gastric gavage to DS-treated and control rats, respectively. To determine the effect of DS supplementation on muscle strength and baseline inflammation, DS supplemented groups were compared with controls in sedentary animals (N = 40) after 10 weeks of DS treatment. To determine the effect of DS supplementation on eccentric exercise-induced muscle damage and inflammatory responses, rats at each dosage were equally split into sedentary (Sedentary, N = 10) and eccentric exercise (EE) groups (N = 10).

The eccentric exercise protocol was modified from previously published methods using downhill running [22]. One week prior to experiments, all animals were acclimated to running on a rat treadmill at 10 m/min for 10 min per day. Rats in the EE group were challenged with an acute bout of intermittent downhill exercise at a 16° decline. Exercises consisted of 18 sessions (5 min/session) separated by 2 min of rest at 16 m/min for a total of 90 min. All rats in EE groups completed the entire exercise session. Two hour after exercise, rats were anaesthetized with intraperitoneal injection of chloral hydrate (400 mg/kg BW). Soleus muscles were excised from both legs and frozen immediately with liquid nitrogen and then stored at −80°C for further analysis.

### Histology and Immunohistochemistry (IHC)

Histology and immunohistochemistry staining were carried out by a pathologist at the Taipei Institute of Pathology (Taipei, Taiwan). Muscle tissues were fixed in formalin and embedded in paraffin. Muscle samples were then sliced to produce 2-µm-thick sections. Sections were stained with hematoxylin and eosin (H&E) to observe tissue histology, and immunohistochemistry was performed to evaluate macrophage invasion and centronucleation as a muscle regeneration marker and to confirm H&E staining [23]. Fixed muscle sections were incubated with mouse antibodies against rat CD68 (dilution 1∶250) (abcam, Cambridge, UK) and rabbit CD163 (dilution 1∶450) (epitomic, San Diego, CA, USA) and then visualized using mouse and rabbit specific HRP/DAB (ABC) detection IHC kit (abcam, Cambridge, UK). Image analysis software Image J (NIH, Bethesda, MD, USA) was used to count the number of CD68- and CD163-positive cells per 100 muscle fibers as described previously [24].

### Western Blotting Analysis

Soleus muscle (∼100 mg) was homogenized in 1 mL of HES buffer containing 20 mM Hepes (Sigma-Aldrich, St Louis, MO, USA), 1 mM EDTA (Sigma-Aldrich, St Louis, MO, USA), and 250 mM sucrose (Sigma-Aldrich, St Louis, MO, USA). Homogenates were centrifuged at 10,000 g for 10 min at 4°C, and the supernatant was collected for Western blotting analysis. Protein (50 µg) was separated by SDS-PAGE (10–12% acrylamide) and transferred to PVDF membranes (PALL Life Science, Ann Arbor, MI, USA) by standard wet transfer methods. After 1 h blocking in PBS containing 5% skim milk, membranes were incubated with primary antibody overnight at 4°C. Primary antibodies against nitrotyrosine were purchased from Millipore (Bedford, MA, USA) (dilution 1∶1000); antibodies against iNOS and eNOS were purchased from BD Transduction Laboratories (Bedford, MA, USA) (dilution 1∶1000); antibodies against p-IKKα/β, IKKα, IKKβ, p-IκBα, IκBα, p-NFκB, NFκB, p38, p-Erk and Erk were purchased from Cell Signaling Technology (Beverly, MA, USA) (dilution 1∶1000); antibodies against p-p38 and GPx (glutathione peroxidase) were purchased from Epitomics (Epitomic, San Diego, CA, USA); antibodies against MnSOD (manganese-containing superoxide dismutase) (dilution 1∶5000) and Cu/ZnSOD (Copper- and zinc-containing superoxide dismutase) (dilution 1∶2000) were purchased from Assay Designs (Ann Arbor, MI, USA); and antibodies against GCS (dilution 1∶100) were purchased from Santa Cruz Biotechnology (Santa Cruz, CA, USA). Antibody-bound protein was detected using a peroxidase-conjugated anti-mouse secondary antibody (Sigma, St Louis, MO, USA) or anti-rabbit IgG (Cell Signaling Technology, Beverly, MA, USA) and enhanced chemiluminescent HRP substrate (PerkinElmer Life and Analytical Sciences, Shelton, CT, USA). To verify equal protein loading, GAPDH was used as an internal control. Western blot bands were quantified using a ChemiDoc XRS+ System (BioRad, Hercules, CA, USA).

### Quantitative Polymerase Chain Reaction (qPCR)

Total RNA was isolated using Qiagen RNeasy Fibrous Tissue Mini Kit (Qiagen, Valencia, CA, USA). Reverse transcription of RNA was carried out using iScriptTM cDNA Synthesis Kit (BioRad, Hercules, CA, USA). The following primer and probe sequences were used for amplification of target genes: COX-2 (NM_017232) forward primer: CAGTCTCTCATCTGCAATA; reverse primer: AGGGTTAATGTCATCTAGTC; probe: TCCCTTTGCCTCTTTCAATGTGC. iNOS (NM_012611) forward primer: TGAGGATTACTTCTTCCAG; reverse primer: TGCTCCATAGGAAAAGAC; probe: CACCGAAGATATCCTCATGATAACGT. TNF-α (NM_012675) forward primer: GAGTCATTGCTCTGTGAG; reverse primer: CTCTGAGGAGTAGACGATA; probe: CTGGCGTGTTCATCCGTTCTCT. IL-6 (NM_012589) forward primer: GAGCAATACTGAAACCCTA; reverse primer: GATGGTCTTGGTCCTTAG; probe: ACTCCTTCTGTGACTCTAACTTCTCCA. IL-1β (NM_031512) forward primer: CCAAGCACCTTCTTTTCC; reverse primer: GTTGGCTTATGTTCTGTCC; probe: CCGTCCTCTGTGACTCGTGG. TaqMan probes contained a FAM reporter at the 5'-end and a TAMRA quencher at the 3'-end. The RT-qPCR conditions have been previously described [25]. Gene expression was normalized to 18S rRNA.

### Wire suspension test

Rat muscle strength was measured by a wire suspension test [26], [27] conducted one night after the final DS treatment. A 12 gauge, 50 cm plastic wire was suspended between two platforms 80 cm above a foam cushioned surface. At the beginning of the test, forepaws were placed on the wire and the time (seconds) to fall was measured in two trials with a 5-minute separation. Muscle strength was defined as maximum time the rat held on to the wire.

### Fiber typing

Staining methods to determine muscle fiber type have been described previously [25]. Briefly, 8 µm thick frozen cross-section of each soleus muscle were sliced at −22°C using Leica CM1900 Cryostat (Leica, Nussloch, Germany). To distinguish between type I and IIa fibers in the soleus, myosin ATPase activity was measured following preincubation at pH 4.55 for 4 min. More than 300 fibers were counted in histochemical sections for quantitation of the fiber ratio.

### Statistical analysis

One-way analysis of variance (ANOVA) was conducted to compare the mean of all variables among groups, with the exception of fiber typing analysis. The Duncan *post hoc* test, which holds the probability value of a type I error to 5 percent for each test, was employed to distinguish differences between pairs of groups. Two-way ANOVA was used to determine the interactive effect of dosage and exercise challenge. Changes in fiber type were analyzed using the non-parametric chi-square method. Variable values of each group are presented as the mean ± standard error (SE). A level of *P*<0.05 was set for statistical significance of difference for all tests.

## Results

**Figure 2:**
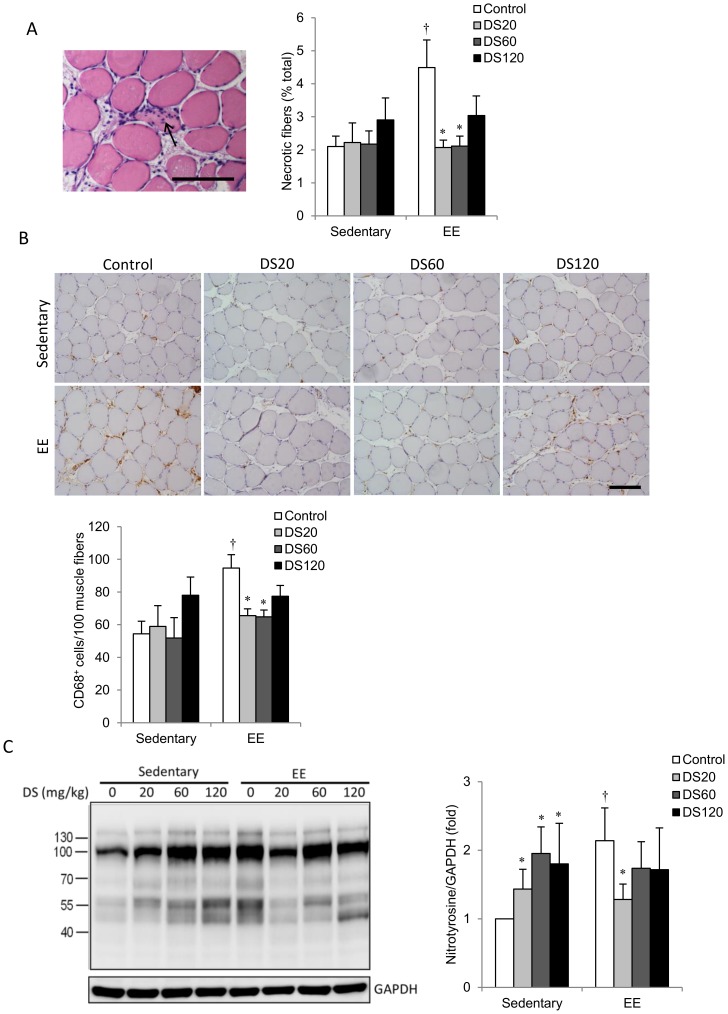
Muscle injury, macrophage invasion and oxidative stress in soleus muscle. (A) Representative histochemical analysis of muscle sections from soleus muscle with H&E staining. Arrowheads indicate immune cell invasion. Scoring is shown on the right. (B) Representative immunohistochemical staining of CD68-positive cells (brown color) in a soleus muscle section. Nucleolus was labeled with eosin staining (blue color). Original magnification was 400x, and scoring of CD68-positive cells is shown on the bottom. (C) Representative western blot showing levels of nitrotyrosine extracted from soleus muscle. Bars represent the relative quantification of nitrated protein normalized to GAPDH. In (A–C), and data are presented as the mean ± SEM. †*p*<0.05 compared with the sedentary group. *p<0.05 compared with the control group of sedentary or EE. Scale bar  = 50 µm.

To evaluate the immune response to EE in skeletal muscle after downhill running, we examined leukocyte invasion and the number of necrotic muscle fibers. H&E staining and immunohistochemical staining for CD68^+^ (M1 macrophages) was performed in rat soleus following 10 weeks of DS supplementation at various doses (Fig. 2). Necrotic muscle fibers and CD68-positive cells were identified by cells invasion (Fig. 2A) and brown color (Fig. 2B), respectively. In non-exercised rats, there was no increase in necrotic muscle fibers (Fig. 2A) or CD68^+^ M1 macrophages (Fig. 2B) in skeletal muscle with DS treatment. However, levels of 3-nitrotyrosine, an oxidative damage marker, increased linearly with dosage (Fig. 2C). Following EE challenge, the number of necrotic muscle fibers increased significantly above the non-exercise control, along with leukocyte and CD68^+^ M1 macrophage infiltration. In rats treated with low and medium doses of DS, such increases were not observed with EE challenge (Figs. 2A and 2B). In saline control rats, 3-nitrotyrosine levels in EE-challenged muscle were significantly greater than in non-exercised muscle. However, this increase was not observed at all doses of DS treatment (Fig. 2C).

**Figure 3:**
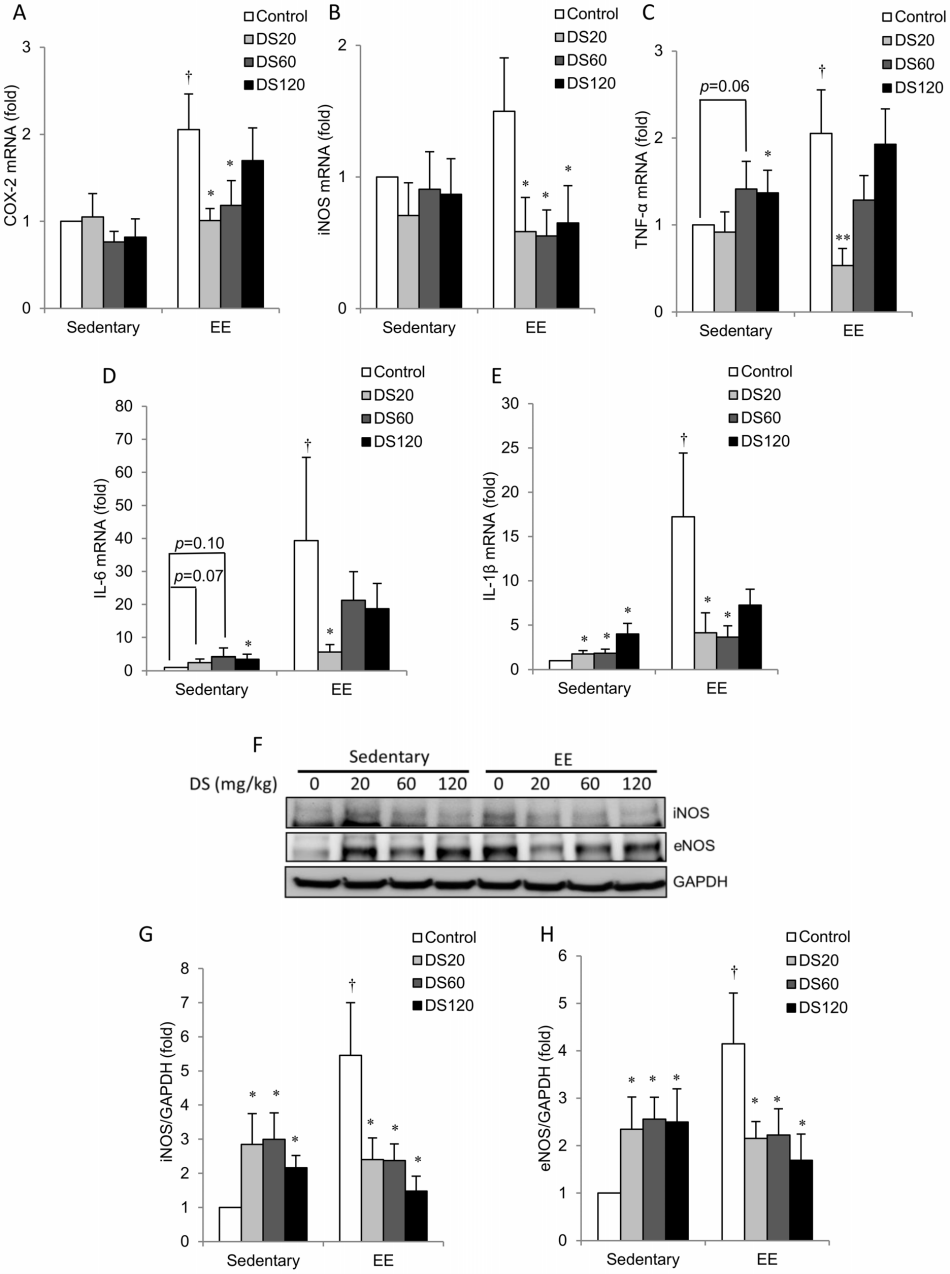
Inflammatory gene and cytokine expression in soleus muscle. (A–E) Bars represent the relative quantification of COX-2, iNOS, TNF-α, IL-6, and IL-1β mRNA expression levels normalized to 18S rRNA. (F) Representative western blot showing protein levels of iNOS and eNOS extracted from soleus muscle. (G–H) Bars represent the relative protein quantification of iNOS (G) and eNOS (H) normalized to GAPDH. In (A–E, G–H), and data are presented as the mean ± SEM. †*p*<0.05 compared with the sedentary group. *p<0.05 compared with the control group of sedentary or EE.

EE-induced inflammatory potentiation was evaluated by measuring mRNA and protein levels of inflammatory mediators in soleus muscle (Fig. 3). In saline control rats, mRNA expression of COX-2, TNF-α, IL-6, and IL-1β (Figs. 3A and 3C–3E) was significantly higher in EE-challenged rats than in sedentary control rats in soleus muscle. iNOS mRNA expression in EE control was marginally higher compared to that in sedentary control rats after EE (Fig. 3B). These responses were attenuated with DS treatment, particularly when low and medium doses of DS were used. Among non-exercise rats, mRNA levels of inflammatory mediators, including TNF-α, IL-6 and IL-1β, increased significantly in the muscle of the DS-treated group in a dose-dependent manner (Figs. 3C–3E). However, expression of inflammatory markers remained below the EE-challenged level (Figs. 3A–3E). Furthermore, EE-challenged rats had increased protein levels of iNOS and eNOS significantly above non-exercise controls in soleus muscle (Figs. 3F–H); these increases were absent in all doses of DS treatment. Among non-exercise control rats, iNOS and eNOS levels were significantly greater in muscle of DS-treated rats than in those of saline control rats; however, levels were lower than those of EE-challenged saline control rats (Figs. 3F–H).

**Figure 4:**
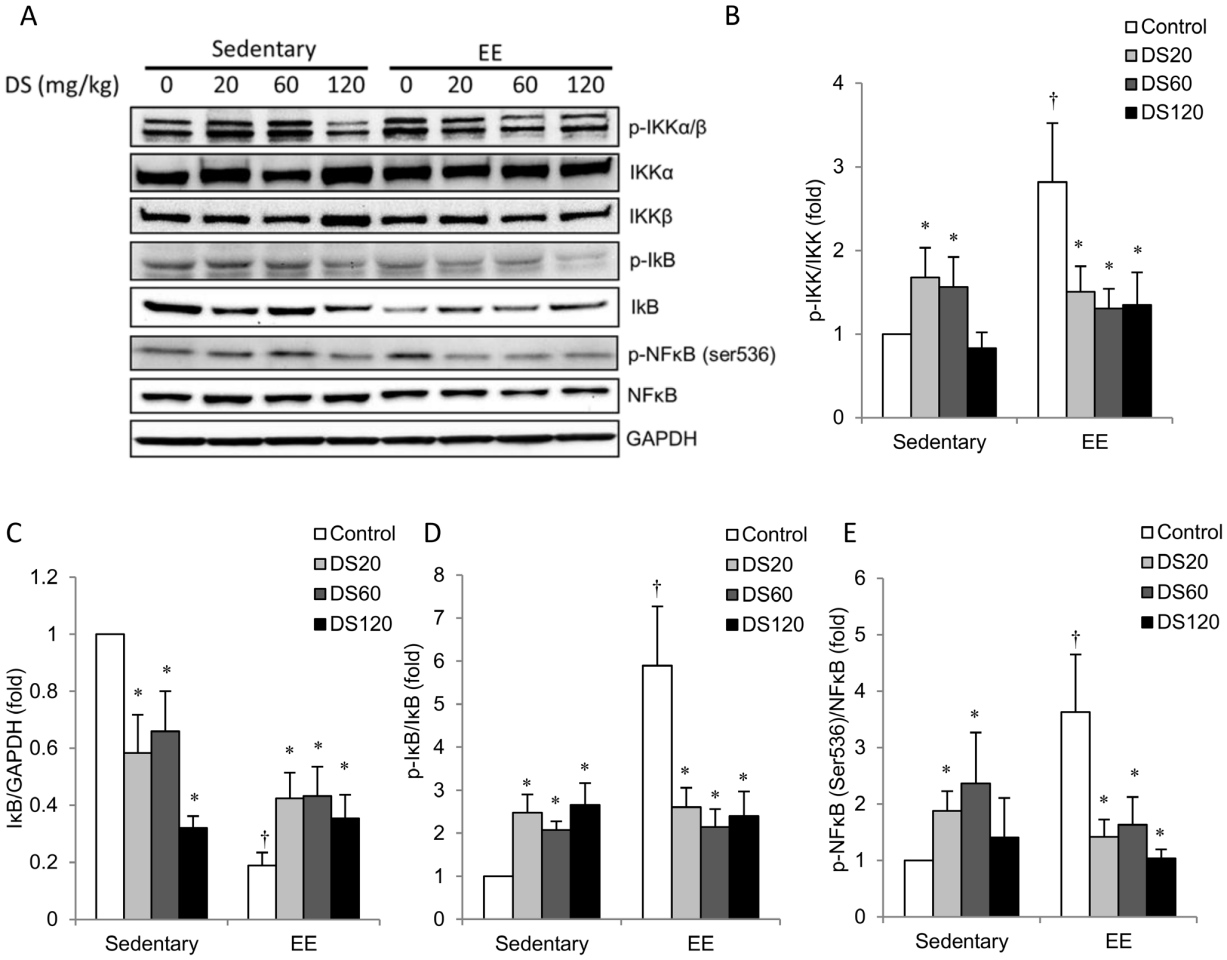
NFκB pathway. (A) Representative western blot showing protein levels of total and phosphorylated IKKα, IκBα and NFκB extracted from soleus muscle. (B–E) Bars represent relative protein quantification of p-IKK/IKK, p-IκBα/IκBα, total IκBα and p-NFκB/NFκB normalized to GAPDH. In (B–E), and data are presented as the mean ± SEM. †*p*<0.05 compared with the sedentary group. *p<0.05 compared with the control group of sedentary or EE.

The NFκB signaling data are shown in Fig. 4. Representative radioactivity is displayed in Fig. 4A using GAPDH as an internal standard. Among saline control rats, p-IKK/IKK (Fig. 4B), p-IκB/IκB (Fig. 4D) and p-NFκB/NFκB (Fig. 4E) levels in muscle of the EE-challenged group were greater than those in the non-exercise control group. IκB was changed in the opposite direction. These changes were attenuated in DS-treated rats at all doses (Fig. 4C). Among non-exercise rats, the 20 mg/kg and 60 mg/kg doses of DS resulted in a significant increase in p-IKK/IKK (Fig. 4B) and p-NFκB/NFκB (Fig. 4E) above saline control levels in muscle. Conversely, DS treatment significantly decreased IκB expression below saline control levels in a dose-dependent manner (Fig. 4C). p-IκB/IκB levels in muscle for all DS-treated rats were significantly greater than those in saline control rats (Fig. 4D).

**Figure 5:**
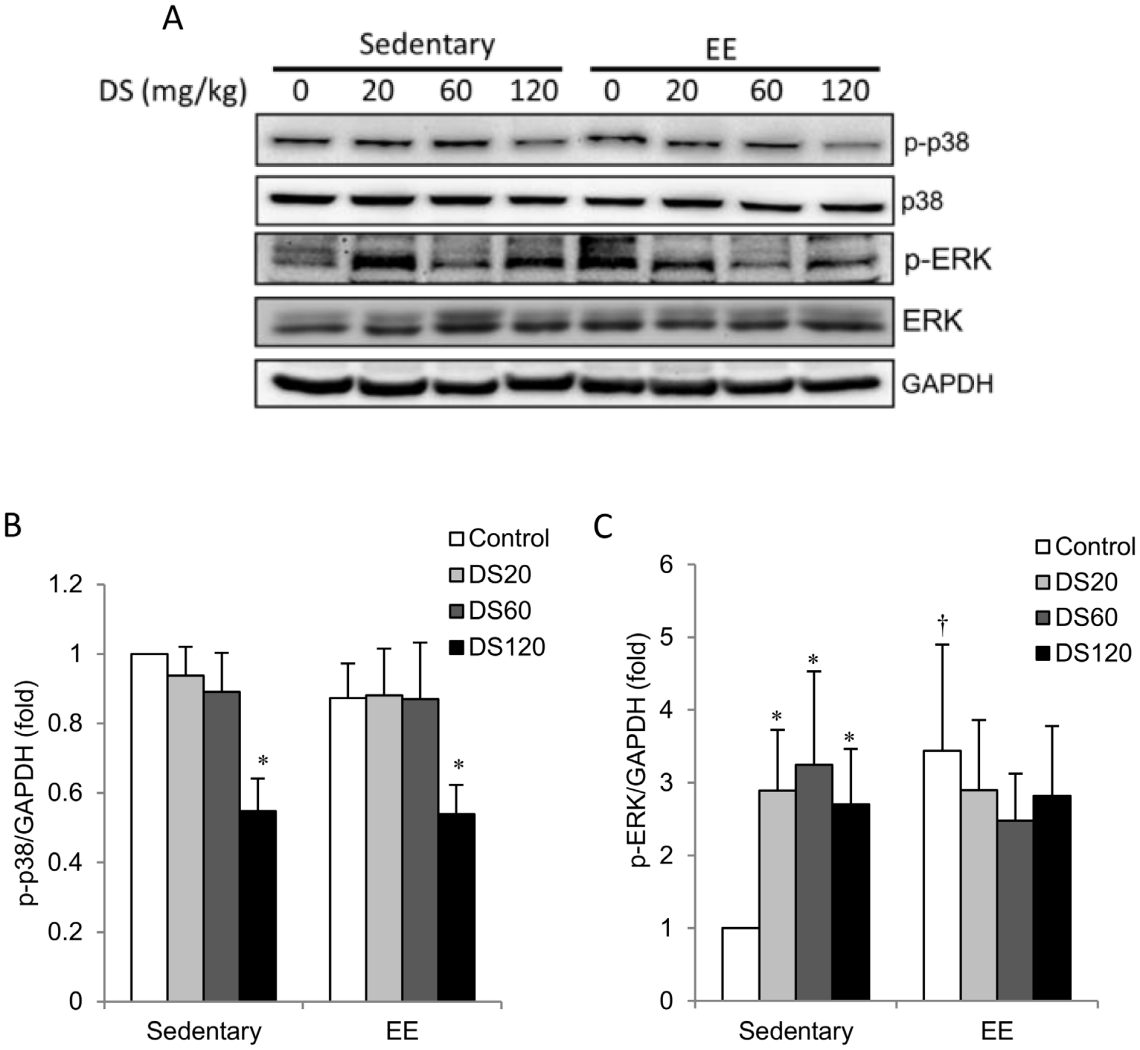
MAPK phosphorylation. (A) Representative western blot showing protein levels of total and phosphorylated p38 and ERK extracted from soleus muscle. (B–C) Bars represent the relative protein quantification of p-p38/p38 and p-ERK/ERK normalized to GAPDH. In (B–C), data are presented as the mean ± SEM. †*p*<0.05 compared with the sedentary group. *p<0.05 compared with the control group of sedentary or EE.

P38 mitogen-activated protein kinases (MAPK) are generally activated by stress-induced inflammation. Here, we found no difference in phosphorylation of p38 MAPK (phospho-p38) between sedentary and EE-challenged muscles (Figs. 5A and 5B). DS treatment at 120 mg/kg resulted in reduced muscle phospho-p38 levels relative to saline controls in both sedentary and EE-challenged rats; however, no apparent change was found at low and medium doses. In contrast, phosphorylation of ERK (phospho-ERK) increased significantly above non-exercise controls in the muscle of EE-challenged rats (Figs. 5A and 5C). All doses of DS supplementation and exercise had similar effects on phospho-ERK levels. No additive effect of DS and exercise on phospho-ERK was observed.

**Figure 6:**
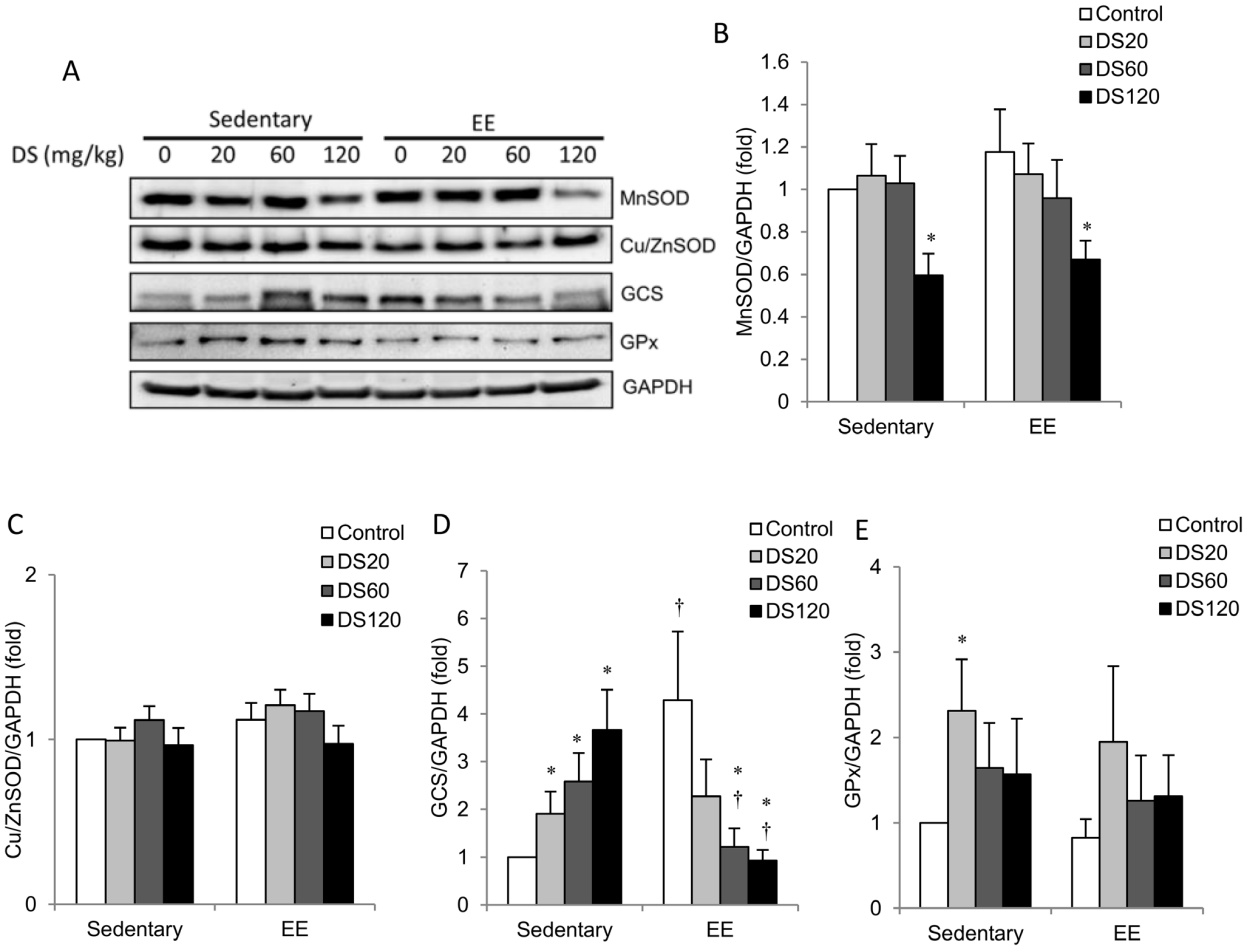
Antioxidant enzymes in soleus muscle. (A) Representative western blot showing protein levels of MnSOD, Cu/ZnSOD, GCS and GPx1 extracted from soleus muscle. (B–E) Bars represent the relative protein quantification of MnSOD (B), Cu/ZnSOD (C), GCS (D) and GPx1 (E) normalized to GAPDH. In (B–E), data are presented as the mean ± SEM. †*p*<0.05 compared with the sedentary group. *p<0.05 compared with the control group of sedentary or EE.

Antioxidant enzymes, including MnSOD, Cu/ZnSOD, GCS and GPx, were evaluated in muscle as shown in Fig. 6. Muscle MnSOD and Cu/ZnSOD were unaffected by EE-challenge (Figs. 6A–6C). MnSOD protein levels in both sedentary and EE-challenged rats were significantly decreased below saline control levels with 120 mg/kg DS treatment (Figs. 6A and 6B). No significant effects of DS on Cu/ZnSOD protein levels were observed (Figs. 6A and 6C). In non-exercise control rats, muscle GCS protein levels were increased with DS treatment in a dose-dependent manner relative to the saline control (Figs. 6A and 6D). Among saline-control rats, EE-challenge resulted in a significant increase in GCS protein levels over sedentary control levels. However, this increase was attenuated in a dose-dependent manner by DS treatment. Only low a dose of DS treatment resulted in increased muscle GPx (Fig. 6E). No effect on GPx was observed with EE-challenge.

**Figure 7:**
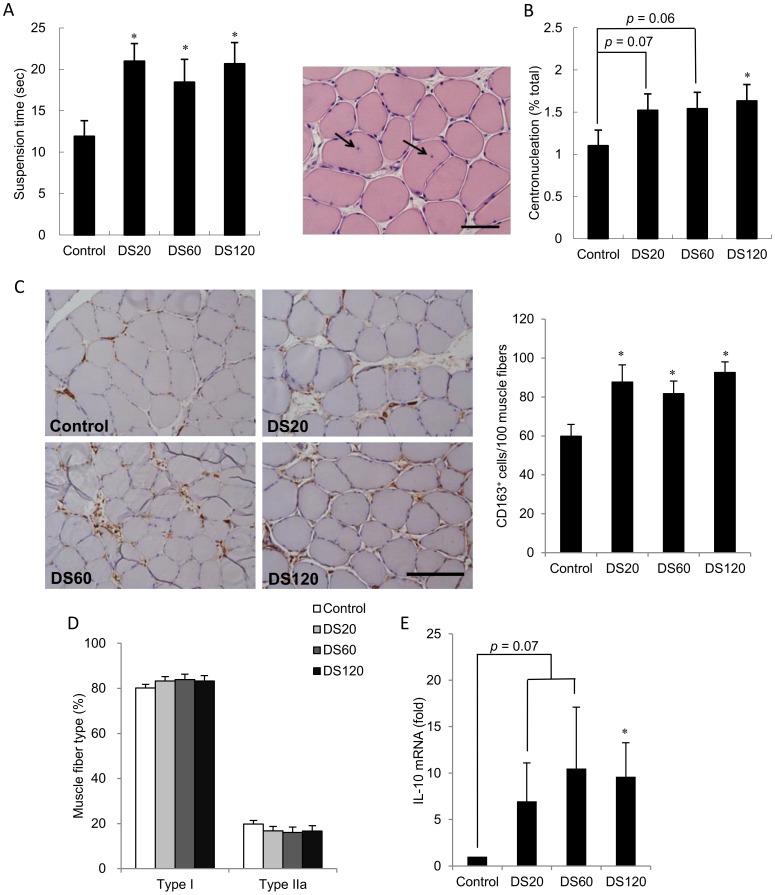
Muscle strength, muscle regeneration, M2 macrophages and muscle fiber types. (A) Bars represent the suspension time (seconds) in the control and 20 mg/kg DS, 60 mg/kg DS, and 120 mg/kg DS groups. (B) Representative histopathological analysis of muscle section from soleus muscle with H&E staining. Arrowheads indicate centronucleation. Scoring of each group is shown on the right. (C) Representative immunohistochemical staining of CD163-positive cells (brown color) in a soleus muscle section. Nucleolus was labeled with eosin staining (blue color). Original magnification was 400x, and scoring of CD163-positive cells is shown on the right. (D) Bars represent the relative quantification of IL-10 mRNA expression levels normalized to 18S rRNA. (E) Bars represent the percentage of muscle fiber types in the control and 20 mg/kg DS, 60 mg/kg DS, and 120 mg/kg DS groups. In (A–E), data are presented as the mean ± SEM. *p<0.05 compared with the control group. Scale bar  = 50 µm.

Muscle strength was evaluated using a wire suspension test (Fig. 7A). Suspension time increased by 40–50% in all doses of DS treatment compared to control rats. Regenerative muscle fibers and CD163-positive cells were identified by centronucleation (Fig. 7A) and brown color (Fig. 7B), respectively. Histological (Fig. 7B) and immunohistochemical (Fig. 7C) analyses show that 10 weeks of DS supplementation increased centronucleation and the number of CD163^+^ M2 macrophages in muscle above saline control level, indicating increased muscle fiber regeneration. Consistent with these data, IL-10 mRNA expression was elevated in DS-treated rats relative to saline controls (Fig. 7D). No effect of DS supplementation on muscle fiber type composition was observed (Fig. 7E).

## Discussion

The major findings of the study are (1) the ginseng-derived steroids DS can potentiate inflammatory signaling of skeletal muscle in a dose-dependent manner, as evidenced by increases in nitrotyrosine levels, NFκB signaling, and gene expression of pro-inflammatory mediators, without apparent muscle fiber injury or CD68^+^ M1 macrophage invasion in skeletal muscle; (2) muscle strength can be increased by long-term DS supplementation at all doses tested; and (3) muscle damage, nitrotyrosine levels, and CD68^+^ M1 macrophage infiltration induced by EE can be minimized at low and medium DS doses, but this protective effect is diminished at a maximal dose. The reverse U-shape dose-dependent outcome suggests that DS have hormetic properties.

Given that muscle fiber composition and muscle mass were not changed, the increased muscle strength in DS-treated rats appears to be associated with increased muscle fiber renewal. DS have been reported to stimulate cell regeneration in skin cells [28], neurons [29], and β cells [30]. Here, we supply additional evidence that increases in muscle strength with DS supplementation are associated with muscle regeneration, evidenced by increased centronucleation. Inflammation is required for muscle fiber turnover in rodents and humans. Pro-inflammatory interventions, such as delivery of myotoxic agents [31] or glucose [32], have been shown to increase muscle regeneration and improve muscle strength. In this study, increased IL-10 mRNA expression and CD163^+^ M2 macrophage localization in skeletal muscle with DS supplementation are consistent with the observed increase in centronucleation. IL-10 is highly expressed in M2 macrophages, which are known to promote muscle regeneration [33], [34]. Taken together, increased cell turnover is expected to reduce average cell age in skeletal muscle, which may underlie the increased resilience of skeletal muscle after long-term DS supplementation.

Though DS supplementation appears to potentiate inflammatory signaling in skeletal muscle, injured fibers and EE-induced oxidative damage were absent in rats with long-term, low-dose DS supplementation. This is consistent with reduced M1 macrophage infiltration, a primary source of NO production during inflammation [4]. M1 macrophage infiltration into tissues typically occurs with cell injury, particularly after traumatic challenge or during cell aging. In early phases of inflammation, phagocytic CD68^+^ M1 macrophages invade and lyse existing injured muscle fibers, followed by a second phase of accumulation of non-phagocytic CD163^+^ M2 macrophages to renew the muscle tissue [7]. Long-term DS supplementation may shorten the length of time required for this muscle regeneration program to produce a younger and healthier muscle fiber population. In previous studies, *Panax ginseng* extract supplementation has been shown to reduce nitric oxide [34] and muscle damage [35] levels in untrained exercise. Our results suggest that DS may be the active component of ginseng that contributes to the putative ergogenic effect reported in previous studies.

In the present study, our data show that DS supplementation significantly increased oxidative stress at rest. Free radicals are a required mediator for perpetuating inflammatory responses and stem cell recruitment [5]. Oxidative stress generated during inflammation amplifies the inflammatory responses mediated by activation of NFκB and MAPK signaling [4] to increase gene expression of pro-inflammatory mediators, e.g., iNOS, eNOS, COX-2 and cytokines [3]. However, EE-induced NFκB/MAPK signaling, M1 macrophage infiltration, and gene expression of inflammatory mediators were attenuated in the skeletal muscle of DS-treated rats. Thus, early renewal of muscle fiber populations by potentiating inflammatory signaling with long-term DS supplementation may explain the protective effect of DS against a muscle-damaging exercise. Whether or not the inflammatory potentiation effects of increasing DS dosage can produce adverse outcome in humans demands further clinical investigation.

The NFκB/MAPK signaling system is also known to enhance expression of antioxidant enzymes [36]. The duration and degree of free radical surge during inflammatory processes can be controlled by alterations in antioxidant levels. The reduction in EE-induced oxidative damage in muscle of DS-treated rats may be explained, in part, by increased expression of antioxidant enzymes, such as GPx and GCS. This is consistent with previous *in vitro* data reporting that ginseng treatment upregulated GPx activity in the soleus muscle of rats [36] and GCS activity in pheochromocytoma PC12 cells [37]. Our data show increased antioxidant enzyme expression and NFκB/MAPK signaling in the muscle of DS-treated rats at low and medium doses; this may explain the attenuated oxidative stress in muscle following EE challenge. However, increased GPx levels were attenuated at a high dose of DS. Additionally, decreased MnSOD levels were observed only with a high dose of DS supplementation; this indicates that too much DS may undermine its protective action. Similar outcomes have also been reported in hyperglycemia-associated chronic inflammation [38]. These results describe the important caveat that different dosages and durations of ginseng use can produce contradictory outcomes in vivo.

Panax ginseng is a popular herbal medicine used worldwide to enhance stamina and coping capacity against physical fatigue. To date, the active ginsenoside component (glycosylated steroids) that contributes to its alleged ergogenic benefit is unknown. Due to its structural similarity to many human steroid hormones, the steroid component of ginseng has been a target of research. However, the steroid profile of ginseng varies with species type and season [20]; this may be a major confounder that contributes to the conflicting results reported in previous ginseng studies [20], [21]. Using ginseng-derived steroids is one way to standardize ginseng and provide a more reliable pharmacological outcomes. The results of this study provide a new perspective and suggest that modulating inflammation with ginseng-derived steroids can influence skeletal muscle performance.

Long-term DS supplementation can potentiate inflammatory signaling in skeletal muscle. However, this treatment can also produce an anti-inflammatory benefit when skeletal muscle is challenged in a muscle-damaging exercise. This result may explain the paradox among previous contradictory findings that described increased cell death and anti-inflammatory activity with DS treatment. Furthermore, the increased muscle strength observed following long-term DS supplementation appears to be associated with decreased cell age of skeletal muscle due to increased cell turnover mediated by inflammatory modulation. The hormetic dose-response relationship of DS suggests that a high dosage should be avoided in clinical applications of ginseng-based supplements.
